# *Biscogniauxia rosacearum* the charcoal canker agent as a pathogen associated with grapevine trunk diseases in Zagros region of Iran

**DOI:** 10.1038/s41598-021-93630-w

**Published:** 2021-07-08

**Authors:** Zeinab Bahmani, Jafar Abdollahzadeh, Jahanshir Amini, Antonio Evidente

**Affiliations:** 1 Department of Plant Protection, Agriculture Faculty, University of Kurdistan, P.O. Box 416, Sanandaj, Iran; 2 Department of Chemical Sciences, University of Naples Federico II, Complesso Universitario Monte S. Angelo, Via cintia 4, 80126 Naples, Italy

**Keywords:** Evolution, Plant sciences

## Abstract

Grapevine trunk diseases (GTDs) are well-known and significant fungal diseases of *Vitis vinifera* with a worldwide distribution. During August to November 2016 in a survey to characterize fungi associated with grapevine trunk diseases in Kermanshah Province (west of Iran) vineyards, 286 fungal isolates were obtained. Based on morphology and DNA sequences data eight species were identified, of which *Biscogniauxia rosacearum*, *Neoscytalidium hyalinum* and *Phaeoacremonium minimum* were the most aggressive fungal pathogenic species characterized in this research. *N. hyalinum* was the most prevalent species. *N. hyalinum* and *Ph. minimum* have previously been reported from *Vitis vinifera*. Thus far, there are two records of *Biscogniauxia mediterranea* and *Biscogniauxia capnodes* on grapevine in the world with no data on pathology aspects. To our knowledge, it is the first time *B. rosacearum* is reported from grapevine across the globe. Pathogenicity test with three strains of *B. rosacearum* on 2-year-old potted grapevines confirmed the pathogenicity of *B. rosacearum* on grapevine. The proximity of vineyards to the oak trees in Zagros forests as one of the plant hosts of *Biscogniauxia* spp. further highlights the need for extensive studies on *B. rosacearum* as a new fungal pathogen.

## Introduction

*Vitis vinifera* L. cultivars are the most widely planted trees around the globe with a high commercial value for fresh table grape, dried fruit, and wine production. According to food and agriculture organization of the United Nations (FAO) statistics, Iran ranks the twelfth in grapevine cultivation with a total area of 165,097 ha in the world, which accounts for 2,032,031 tons annual production^1^. The worldwide economic cost for the replacement of dead grapevines is roughly estimated to be in excess of 1.5 billion dollars per year^2^. Among biotic stresses fungal pathogens have a significant negative effects on the quantity and quality of grapevine. Grapevine trunk diseases (GTDs) are the most destructive grapevine diseases which threaten the grape production all around the world^3^. The main grapevine trunk diseases are known as esca complex, eutypa and botryosphaeria dieback and excoriosis^4^. For many years, *Eutypa lata* has been considered as the main fungal species involved in grapevine canker and decline disease worldwide^4^. However, intensive studies undertaken on grapevine trunk disease in last two decades in Iran and other countries worldwide have revealed that in addition to *Eutypa*, several other ascomycetes including species of the genus *Phaeoacremonium*, *Phaeomoniella*, *Phomopsis*, members of the family *Botryosphaeriaceae* (e.g. *Botryosphaeria*, *Diplodia*, *Lasiodiplodia*, *Neofusicoccum*), *Diatrypaceae* (e.g. *Cryptovalsa*, *Diatrype* and *Eutypella*) and *Didymosphaeriaceae* (e.g. *Paraconiothyrium*) are associated with trunk diseases of grapevines^3,5–13^. More recently, other fungal groups such as *Acremonium*, *Cadophora*, *Cytospora*, pestalotioid and *Phoma*-like fungi, have also been reported in association with grapevine trunk diseases, which have proven to be pathogenic on grapevine in pathogenicity assays^14–16^.

*Biscogniauxia* Kuntze in the family *Graphostromataceae* (*Xylariales*) encompasses more than 100 species (Nov. 2020; www.indexfungorum.org, www.mycobank.org) reported from a broad spectrum of woody hosts around the world such as species of *Acacia*, *Acer*, *Alnus*, *Amygdalus*, *Casuarina*, *Citrus*, *Corylus*, *Eucalyptus*, *Fagus*, *Fraxinus*, *Juglans*, *Juniperus*, *Malus*, *Mangifera*, *Pinus*, *Populus*, *Prunus*, *Pyrus*, *Quercus*, *Ribes*, *Rubus*, *Salix*, *Sorbus*, *Ulmus* and *Vitis* as retrieved from https://nt.ars-grin.gov/fungaldatabases/ at Nov. 2020^17^. Of these, only two species of *Biscogniauxia capnodes* and *Biscogniauxia mediterranea* have been reported from *Vitis* in Taiwan and United States^17^.

Typically, species of *Biscogniauxia* are found as endophytes and opportunistic pathogens on old and stressed trees. So far five species; *Biscogniauxia anceps* (on *Diospyros lotus*, *Mespilus germanica*, *Parrotia persica*, *Quercus castaneifolia*), *B. capnodes* (on *Quercus castaneifolia*), *Biscogniauxia mediterranea* (on *Amygdalus scoparia*, *Quercus brantii*, *Quercus castaneifolia*), *Biscogniauxia plana* (on *Diospyros lotus*, *Quercus castaneifolia*) and *Biscogniauxia rosacearum* (on *Quercus castaneifolia*) have been reported from Iran^18–23^. The most important species *Biscogniauxia mediterranea*, the causal agent of charcoal canker of oak trees, is a well-known and serious threat for Zagros oak forests, west of Iran^22,23^. *B. rosacearum* characterized as a closely related species to *B. mediterranea* on seven different forest and fruit trees from Iran, Italy, Portugal and Spain^20^. They confirmed the pathogenicity of *B. rosacearum* and recorded the charcoal canker symptom caused on pear, plum and quince trees. Here we report *B. rosacearum* from vineyards showing grapevine trunk diseases symptoms and confirm pathogenicity of this species on *V. vinifera* for the first time. Based on our knowledge after *B. capnodes* from Taiwan, *B. rosacearum* is the second species of *Biscogniauxia* that is reported here from *Vitis vinifera*.

## Results

### Fungal isolation and molecular identification

In this survey 286 fungal isolates were obtained from the wedge-shape and central brown necrosis of wood observed in cross-sections of infected cordons and trunks. Based on morphological studies fungal isolates were divided to 12 morphological groups identified as *Alternaria*, *Aspergillus*, *Biscogniauxia*, *Cladosporium*, *Curvularia*, *Cytospora*, *Neoscytalidium*, *Penicillium*, *Phaeoacremonium*, Pestalotia-like, Phoma-like and a phialidic group. We didn’t further considered isolates identified as *Alternaria* (28 isolates/9.8%), *Aspergillus* (10 isolates/3.5%), *Cladosporium* (16 isolates/5.6%), *Cytospora* (9 isolates/3.1%) and *Penicillium* (11 isolates/3.8%). Based on colony morphology, cultural characteristics, microscopic features and geographic location 78 fungal isolates belong to *Biscogniauxia* (11 isolates), *Curvularia* (5 isolates), *Neoscytalidium* (17 isolates), *Phaeoacremonium* (10 isolates), Pestalotia-like (10 isolates), Phoma-like (20 isolates) and phialidic group (5 isolates) were selected for molecular studies. To reduce the number of isolates for sequencing all 78 isolates were grouped based on the ISSR fingerprinting patterns generated with M13 primer (data not shown) and a representative isolate for each group selected for phylogenetic analyses and pathogenicity tests.

Based on morphology, nucleotide blast and phylogenetic analyses the representative isolates were belong to eight species namely *Biscogniauxia rosacearum*, *Clonostachys rosea*, *Curvularia spicifera*, *Kalmusia variispora*, *Microsphaeropsis olivacea*, *Neoscytalidium hyalinum*, *Phaeoacremonium minimum* and *Truncatella angustata*. Of these, *Neoscytalidium hyalinum* with 51 isolates from 11 vineyards (18%) was the most prevalent identified species. *B. rosacearum* with 16 isolates from 2 vineyards (5.6%) was the seventh species identified for the first time in association with grapevine trunk diseases around the world. The number of isolates, frequency and geographic location of the identified species are presented in Table 1.

**Table 1:** Isolates number, frequency, geographic location and culture collection and GenBank accession numbers of fungal species identified in this study.

Species	Isolates/vineyard no	Frequency (%)	Geographic location	Sequenced isolates	GenBank accession number		
					ITS	*tef-1α*	*tub2*
*Biscogniauxia rosacearum*	16/2	5.6	Paveh	IRAN 4193C IRAN 4194C IRAN 4195C	MW786619 MW786620 MW786621	– – –	– – –
*Clonostachys rosea*	15/2	5.2	Soomar	CJAZBKJK1	MW820086	–	–
*Curvularia spicifera*	19/3	6.6	Javanrud, Kerend–e Gharb	CJAZBMR	MW820087	–	–
*Kalmusia variispora*	33/6	11.5	Dinavar, Javanrud, Paveh, Soomar	CJAZBGHC	MW820088	–	–
*Microsphaeropsis olivacea*	16/3	5.6	Soomar, Harsin	CJAZBASK1	MW820089	–	–
*Neoscytalidium hyalinum*	51/11	18	Dinavar, Harsin, Kerend-e Gharb, Paveh, Soomar	CJAZBMAF	–	MW856466	–
*Phaeoacremonium minimum*	24/3	8.4	Javanrud	CJABZSBK1	–	–	MW856465
*Truncatella angustata*	38/6	13.3	Dinavar, Javanrud, Paveh	CJAZBNRK1	MW820090	–	–

Since *Biscogniauxia rosacearum* the causal agent of the charcoal canker of trees is reported on *V. vinifera* showing decline and wedge-shape necrosis of the wood as a new record worldwide the ITS phylogeny with three representative isolates is presented.

### ITS phylogeny of *B. rosacearum*

**Figure 1:**
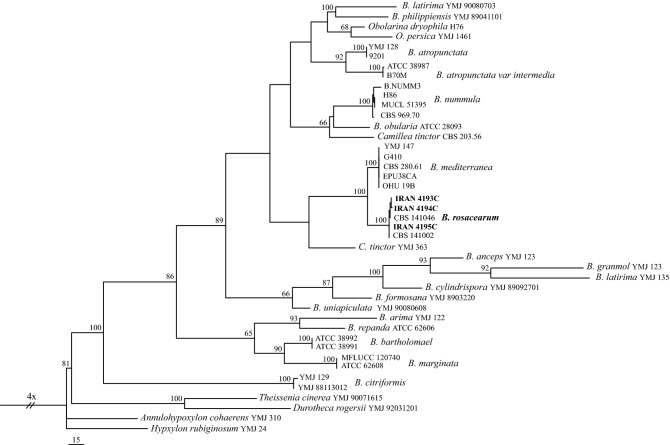
One of the two most parsimonious trees obtained from analysis of ITS sequence data. MP bootstrap values based on 1000 pseudoreplicates are above the branches. The isolates and species characterized in this study are in boldface. Bar = 15 changes. *Annulohypoxylon cohaerens* YMJ 310 and *Hypoxylon rubiginosum* YMJ 24 were included as outgroups.

ITS sequences of three isolates sequenced in this study together with 38 sequences of 24 *Biscogniauxia* species and two outgroups (*Annulohypoxylon cohaerens* YMJ 310/*Hypoxylon rubiginosum* YMJ 24) retrieved from GenBank were aligned and subjected to the maximum parsimony analysis. After alignment the dataset consisted of 768 characters including alignment gaps. Of these, 232 were constant, 130 were variable and parsimony-uninformative and 406 were parsimony-informative. Maximum parsimony analysis of the 406 parsimony-informative characters resulted in two most parsimonious trees (TL = 2124, CI = 0.53, RI = 0.71, HI = 0.47). Our isolates all clustered in a clade containing ex-type strain of *B. rosacearum* (Fig. 1).

### Pathogenicity

**Figure 2:**
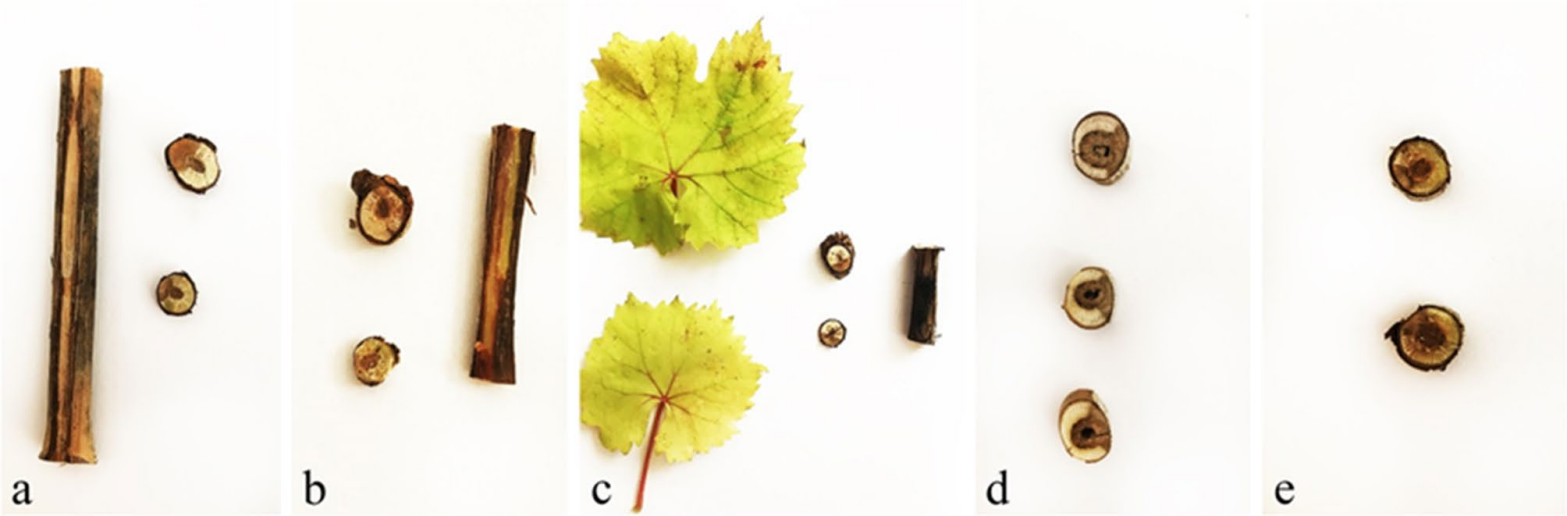
Disease symptoms caused by pathogenic fungi on grapevines in greenhouse conditions. (**a**) *Curvularia spicifera*. (**b**) *Kalmusia variispora*. (**c**) *Neoscytalidium hyalinum*. (**d**) *Pheoacremonium minimum*. (**e**) *Truncatella angustata*.

**Figure 3:**
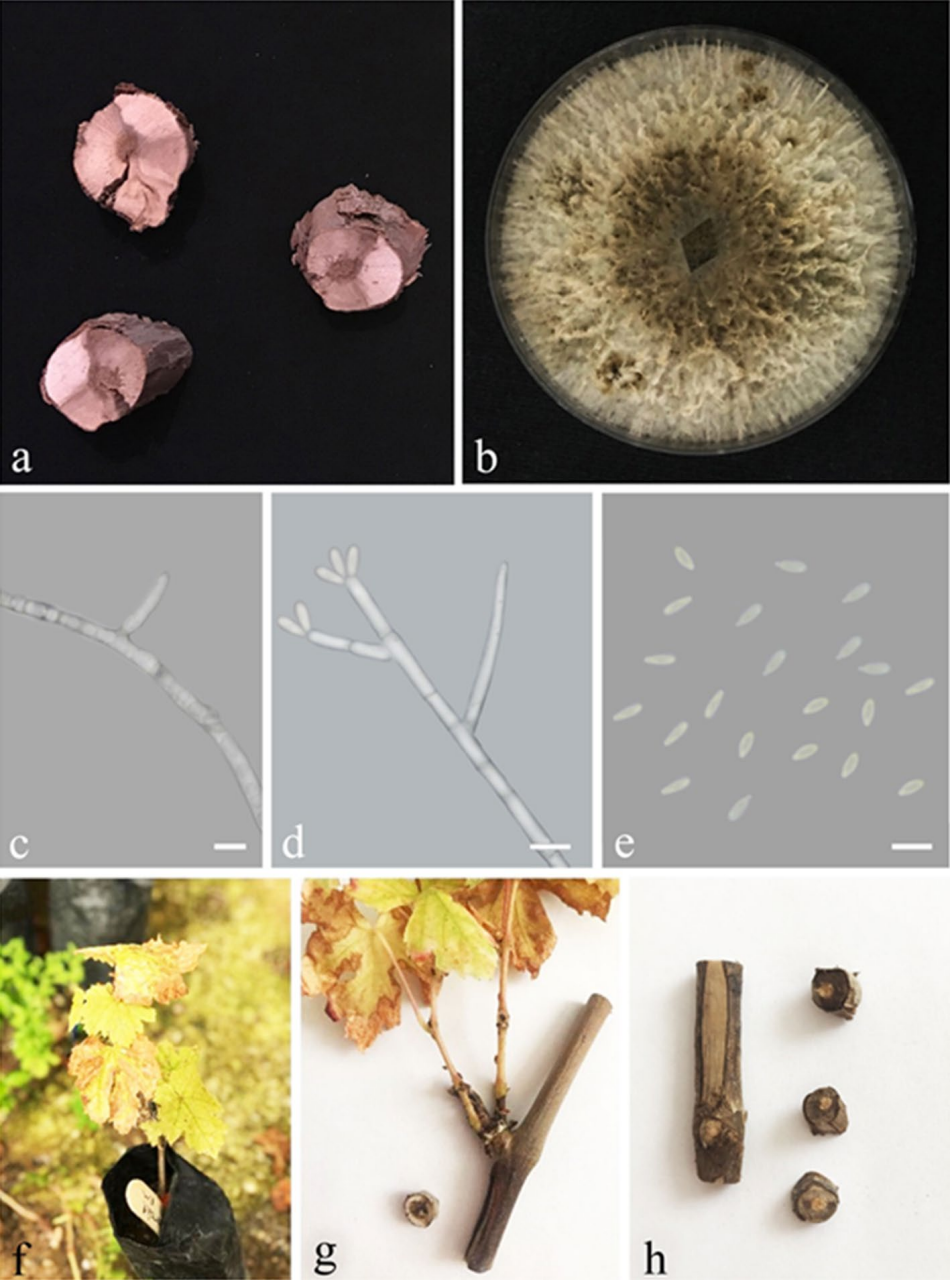
*Biscogniauxia rosacearum* colony, asexual morphology and disease symptoms on grapevine. (**a**) wedge-shape brown necrosis of wood in cross-sections of trunks and cordons from which *B. rosacearum* was isolated. (**b**) seven-day-old colony on MEA at 25 °C. (**c,d**) conidiophores and phialides. (**e**) conidia. (**f–h**) inoculated plant after six weeks, chlorosis and necrosis on leaves and wedge-shape brown necrosis of wood in cross-sections of shoots in greenhouse conditions. Bars = 7 μm.

Among the eight identified species *Clonostachys rosea* and *Microsphaeropsis olivacea* were non-pathogenic and the other six species were pathogenic. All pathogenic species caused yellowing and necrosis of leaves and wedge-shape brown necrosis of wood in cross-sections of inoculated shoots in greenhouse condition (Figs. 2 and 3a,f–h). All inoculated fungi were successfully re-isolated from brown wedge-shape lesions and Koch’s postulates confirmed. Disease symptoms did not observed on control plants and no fungi were re-isolated.

*Biscogniauxia rosacearum*, *Neoscytalidium hyalinum* and *Phaeoacremonium minimum* were the most aggressive species and showed high virulence on 2-year-old potted grapevine (cv. Bidaneh sefid) under greenhouse conditions followed by *Truncatella angustata*, *Kalmusia variispora* and *Curvularia spicifera*.

It is the first time that *B. rosacearum* is confirmed as pathogenic and reported as a new fungal pathogen on grapevine. In pathogenicity assay of *B. rosacearum* disease symptoms including yellowing and necrosis of leaves were appeared 14 days after inoculation in greenhouse conditions. Brown lesions expanded upwards and downwards of the inoculation point on the stem internodes and stem cross-sections showed wedge-shape necrosis of the vascular tissue (Fig. 3f–h).

## Discussion

As mentioned above many fungal species have been identified in association with grapevine trunk diseases (GTDs). In this study we identified eight fungal species associated with grapevine trunk diseases in Kermanshah Province vineyards of which *Clonostachys rosea* and *Microsphaeropsis olivacea* were found non-pathogenic species in greenhouse conditions. *C. rosea* has been reported as pathogen, saprophyte and endophyte and in association with wood decay and root rot of grapevine in China and Switzerland^17^ and here it is reported as a non-pathogenic species in association with grapevine trunk diseases for the first time from Iran. *Biscogniauxia rosacearum*, *Neoscytalidium hyalinum* and *Phaeoacremonium minimum* were the most aggressive pathogenic species in greenhouse conditions. *Ph. minimum* is a well-known fungal pathogen associated with grapevine trunk diseases all around the world. *N. hyalinum* has been reported as the causal agent of dieback and wood canker in association with grapevine trunk diseases in Brazil, India, Iraq, Turkey and California^17^, whereas *B. rosacearum* is a new pathogen with high virulence on grapevine in greenhouse conditions.

*Biscogoniauxia* species are not among fungal species associated with grapevine trunk diseases and we found only two studies that introduced *Biscogoniauxia* on *Vitis*^17^. *B. capnodes* and *B. mediterranea* have been reported on *Vitis vinifera* from Taiwan and *Vitis* sp. from United States, respectively^14,24^. In this survey based on ITS sequence data we introduce another *Biscogoniauxia* species, *B. rosacearum* a closely related species to *B. mediterranea* on *Vitis* in association with grapevine trunk diseases. *B. rosacearum* was described as a new species from Iran (*Q. castaneifolia*), Italy (*Cydonia oblonga*/*Prunus domestica*/*Pyrus communis*/*Quercus pubescens*), Portugal (*Platypus cylindrus*/arthropods) and Spain (*Holcus lanatus*/*Quercus ilex*/*Pinus sylvestris*)^20^. They confirmed pathogenicity of *B. rosacearum* on pear, plum and quince^20^. In this study it was pathogenic and virulent on grapevine in greenhouse conditions. Inoculated plants showed decline symptoms including chlorosis and necrosis on leaves and wood necrosis and browning on stems. Here we introduce *B. rosacearum* as a new pathogen in association with grapevine trunk diseases. The symptoms induced by *B. rosacearum* on grapevine and other hosts indicate that phytotoxins are significant pathogenicity weapon in this fungal pathogen. So far several phytotoxins from pathogenic fungi associated with grapevine trunk diseases have been characterized and their role in the development of disease symptoms has recently been discussed^25–27^. The only phytotoxins isolated from *Biscogniauxia* are the new phytotoxic hexasubstituted pyranopyran isolated from *B. mediterranea*, a major fungal pathogen involved in oak decline^28^. Thus, it is important to characterize biological and chemical properties of the phytotoxins produced by *B. rosacearum*.

Given that *B. rosacearum* has recently been introduced very little is known on the extent of distribution, host range and mechanisms of dispersal of this fungus in Zagros region and other part of the country. Since most of *Biscogniauxia* species has been reported on oak trees in various part of the world^15^ and *B. mediterranea* the close species to *B. rosacearum* has reported as a serious threat for oak trees in Zagros region^22,23^, it is important to consider close cultivation of grapevine to oak forests in Zagros region that may have inherent risks in terms of the availability and dispersal of viable inoculum between vineyards and oak forests. Thus, it is a necessity to focus on pathogenicity of this species on different grapevine cultivars and fruit and forest trees especially oak species in Zagros forests, population biology of this fungal pathogen including epidemiology and population genetics, and integrated disease management consisting a combination of strategies and tactics including biological agents, sanitation, resistant genetic resources, pruning, etc. and chemical fungicides if necessary.

## Materials and methods

### Symptoms and fungal isolation

During August to November 2016, 90 samples were collected from trunks and cordons of own rooted grapevines showing yellowing, dieback, esca and decline symptoms from 19 vineyards (> over 10 years old) of Kermanshah Province (a part of Zagros region in west of Iran). Small pieces of infected woods showing necrosis were plated out after surface sterilization onto potato dextrose agar (PDA) plates supplemented with 100 mg/L streptomycin sulphate and ampicillin. Pure cultures were prepared using single-spore technique.

### Morphological studies

All collected fungal isolates were studied morphologically and grouped based on colony morphology, cultural characteristics and fungal structures morphology including conidiomata, conidiophores, conidiogenous cells and conidia. Fungal isolates were grown on PDA, malt extract agar (MEA), oat meal agar (OA) or pine needle agar (PNA). If needed to induce sporulation fungal isolates were incubated under mixed white and black light in a 12 h light–dark regime. Fungal structures were mounted in 100% lactic acid or water. Morphological features were recorded with an Olympus DP72 camera on an Olympus BX51 microscope. Measurements were made with the Cell Sense Entry measurement module. Dimensions of each fungal structure were calculated based on at least 30 microscopic measurements.

### DNA extraction and sequencing

Genomic DNA of pure cultures was extracted according to reported method as previously described^29,30^. To identification of fungal isolates at the species level different gene regions (ITS, *TEF-1α* and *TUB2*) were sequenced depending the genera or fungal taxonomic groups inferred based on morphology. The PCR reaction mixtures in 25 µL volume prepared containing 1 × PCR buffer, 3 mM MgCl2, 200 µM of each nucleotide, 5 pmol of each primer, 1 U of *Taq* polymerase and 1 µL of template DNA (50–100 ng/µL). The PCR conditions were: primary denaturation step of 5 min at 96 °C followed by 35 cycles of 30 s at 94 °C, 30 s at 52 °C (ITS and *TEF-1α*) or 58 °C (*TUB2*) and 1 min at 72 °C, with a final extension of 7 min at 72 °C. The PCR products were purified and sequenced by Macrogen Co. (South Korea). Consensus sequences were obtained using the software BioEdit v. 7.0.0^31^.

### Molecular identification and phylogenetic analysis of fungal isolates

The generated sequences were blasted against the National Center for Biotechnology Information (NCBI) GenBank sequence database using MegaBLAST to identify related taxa at the genus level. To identification at the species level sequences were subjected to maximum parsimony (MP) phylogenetic analyses using PAUP v. 4.0b10^32^.

Generated sequences and authentic sequences retrieved from GenBank together with the outgroup species were aligned using ClustalX v. 1.83^33^. Alignments were edited manually where necessary. For phylogenetic analysis a maximum parsimony analysis was performed in PAUP v. 4.0b10^32^. The heuristic search option with 1000 random taxa additions and tree bisection and reconnection (TBR) branch-swapping algorithm were used. All characters were unordered and of equal weight. Gaps were treated as missing data. Branches of zero length were collapsed and all multiple, equally parsimonious trees were saved. The robustness of the most parsimonious trees was evaluated by 1000 bootstrap replications^34^. Other measures used were consistency index (CI), homoplasy index (HI), retention index (RI) and tree length (TL).The generated equally most parsimonious phylogenetic trees were visualized in FigTree v. 1.4.3 (http://tree.bio.ed.ac.uk/software/figtree) and edited in Adobe Illustrator CS2 v. 12.0.0.

### Pathogenicity tests

Pathogenicity tests were carried out using 7-day-old colonies of fungal isolates on 5 mm diam. wounds on the stems internodes of 2-year-old potted healthy grapevine (cv. Bidaneh Sefid) under greenhouse conditions. Inoculated wounds were wrapped by Parafilm. The stems of control plants were inoculated with sterile agar plugs. The bark was removed and lesion lengths were measured after 6 weeks. Koch’s postulates was confirmed by plating out infected tissues on PDA to recover inoculated fungal isolates. The use of plant parts in the present study complies with national guidelines.
